# Width of pubic symphysis relating to age and sex in Koreans

**DOI:** 10.1186/s13018-021-02561-9

**Published:** 2021-07-03

**Authors:** Kun Hwang, Xiajing Wu, Chan Yong Park

**Affiliations:** 1 Department of Plastic Surgery, Inha University College of Medicine, 27 Inhang-ro, Jung-gu, Incheon, 22332 Republic of Korea; 2 Division of Trauma Surgery, Department of Surgery, Seoul National University Hospital, 101 Daehak-ro, Jongno-gu, Seoul, 03080 Republic of Korea

**Keywords:** Pubic symphysis, X-ray film, Anatomy, Cross-sectional, Pubic symphysis diastasis

## Abstract

**Introduction:**

Diastasis of the pubic symphysis has been reported to occur in 13–16% of pelvic ring injuries. In Asians, there are only a few data showing the width of the pubic symphysis. The aim of this study is to see the width of pubic symphysis relating to age and sex in Koreans.

**Methods:**

Width of pubic symphysis was measured in pelvis AP and pelvic CT of 784 peoples (392 males, 392 females).

**Results:**

In supine AP, the width at the upper end was 4.8±2.5 mm (males; 3.46±1.38 mm, females; 4.04±2.76 mm). The width at the midpoint was 4.7±2.0 mm (males; 4.64±1.58 mm, females; 4.75±2.29 mm). The width at the lower end was 4.8±2.5 mm (males; 4.58±2.19 mm, females; 5.08±2.76 mm). In abducted AP, the width at the upper end was 3.8±2.9 mm (males; 3.65±1.50 mm, females; 3.97±3.85 mm). The width at the midpoint was 4.6±2.3 mm (males; 4.45±2.16 mm, females; 5.18±3.79 mm). The width at the lower end was 4.8±3.1 mm (males; 4.55±1.30 mm, females; 4.74±3.06 mm). In axial CT, the width at the anterior border was 15.0±6.2 mm (males; 14.50±6.62 mm, females; 16.44±6.22 mm). The width at the narrowest point was 3.1±1.5 mm (males; 3.19±1.53 mm, females; 3.09±1.50 mm). The width at the widest point was 4.1±1.6 mm (males; 4.27±1.60 mm, females; 4.00±1.50 mm). The width at the posterior border was 2.3±1.3 mm (males: 2.20±1.30 mm, females; 2.44±1.40 mm). Axial thickness was 27.1±5.3 mm (males; 29.48±4.60 mm, females; 24.70±4.82 mm). In coronal CT, the width at the upper end was 3.1±4.1 mm (males; 2.28±1.26 mm, females; 3.83±5.48 mm). The width at beginning of widening was 3.6±4.5 mm (males; 2.68±1.63 mm, females; 4.54±6.08 mm). The width at the lower end was 20.5±8.2 mm (males; 17.49±4.53 mm, females; 23.60±9.86 mm). Coronal thickness was 20.4±7.1 mm (males; 24.50±5.98 mm, females; 16.23±5.61 mm). In supine film, width significantly increased with age at the upper end (p=0.022) and midpoint (p< 0.001); however, it decreased at the lower end (p< 0.001). In abduction film, width at midpoint increased with age (p=0.003).

**Conclusion:**

Pelvic malunion should be defined according to the population and age. These results could be a reference in assessing the quality of reduction after internal fixation of the patients with traumatic diastasis of the pubic symphysis.

**Supplementary Information:**

The online version contains supplementary material available at 10.1186/s13018-021-02561-9.

## Introduction

Diastasis of the pubic symphysis is one type of pelvic injury and has been reported to occur in 13–16% of pelvic ring injuries and occur following a high-velocity force such as road traffic accidents and particularly in those involving motorcyclists, horse riding, crush injuries, and falls from a height [1, 2].

Techniques for managing traumatic diastasis of the pubic symphysis include bed rest, hip spica casting, pelvic slings, external fixation, and internal fixation [3].

The common hardware complications are infections, loosening, small particle disease/osteolysis, periprosthetic fracture, hardware fracture or dislocation, and recurrent disease, especially in patients with tumors [4]. Assessing the quality of reduction, fixation failure has been defined as either plate/screw loosening or breakage that resulted in a loss of postoperative reduction. Anatomically, the adductor longus and rectus abdominis are attached to the capsule and disk of the symphysis pubis which causes the pubic diastasis in injury [5]. The pelvic malunion has been defined as greater than 5-mm of displacement of the hemipelvis and pubic symphysis in a nonanatomic position, whether in a rotational or translational fashion [6].

Measuring adult cadavers, Loeschcke (1912) calculated mean joint widths to be 5 mm in men, 7.5 mm in nulliparous women, and 20 mm in multiparous women, but precise details of how these measurements were taken are lacking [7].

In Asians, however, there are only a few data showing the width of pubic symphysis [8, 9]. Since the cartilage is removed in internal fixation, difficulties remain in assessing the quality of reduction after internal fixation of the patients with traumatic diastasis of the pubic symphysis.

We thought if we could show the changes in pubic symphysis width in distinct age- and gender-dependent plots, they could serve as standards of comparison to detect pathologic or posttraumatic changes in each age and sex group.

The aim of this study is to see the width of pubic symphysis relating to age and sex in Koreans.

## Materials and methods

### Materials

From 2003 to 2016, retrospective reviews of plane pelvis AP and CT of subjects were done on subjects without recent trauma to the pelvis. Any subjects who had prior surgery, radiotherapy of the contra-lateral healthy area, inflammation, infection, and/or a tumor were excluded. The selected 784 CT images (392 Korean males, 392 Korean females, age ranged 0 to 99, 0–10 years: 2 subjects, 11–20 years: 15 subjects, 21–30 years: 77 subjects, 31–40 years: 85 subjects, 41–50 years: 109 subjects, 51–60 years: 144 subjects, 61–70 years: 135 years, 71–80 years: 140 subjects, 81–90 years: 64 subjects, 91–100 years: 13 subjects, mean age 56.6±18.9 years) were analyzed (Table 1).

**Table 1:** Number of males and females in 10 years intervals

Age (years)	N		
	Male	Female	Total
0–10	0	2	2
11–20	9	6	15
21–30	54	23	77
31–40	46	39	85
41–50	50	59	109
51–60	82	62	144
61–70	76	59	135
71–80	53	87	140
81–90	16	48	64
91–100	6	7	13
Total	392	392	784

The radiological images were obtained from an electronic image repository of Inha University Hospital, Incheon, Korea.

### Standard process taking images

For Pelvis AP in abduction, both femurs were abducted 60 degree and knees were flexed to face each sole together in supine position. Position was held to have symmetrical obturator foramens and iliac crests. For coronal and axial CT of the pubic symphysis, subjects were laid in supine position with both anterior superior iliac spines in the same level. Coronal view includes from the 4th lumbar spine to the lesser trochanter of the femur. The DICOM files from the electronic image repository were used. Measurements were not performed in 504 samples of abduction AP, 286 of axial CT, and 8 of coronal CT.

### Measuring methods

**Fig. 1:**
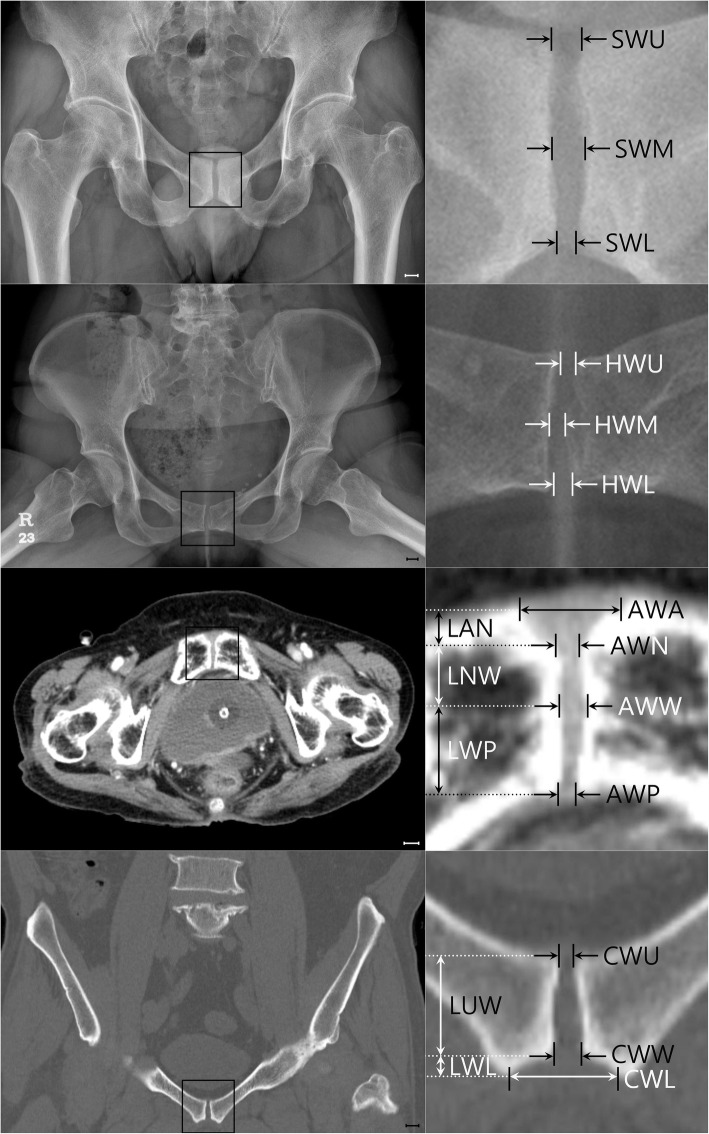
Measurement of the width of the pubic symphysis. First raw: pelvis AP (supine), second raw: pelvis AP (hip abduction view), third raw: pelvic CT (axial view), fourth raw: pelvic CT (coronal view). The scales in the left columns indicate 1 cm each

Two researchers measured the width of the pubic symphysis in pelvis AP and pelvis CT (Fig. 1).

#### Pelvis AP (supine)

1. Width of pubic symphysis at the upper (cranial) end (SWU)
2. Width of pubic symphysis at the midpoint of the upper and lower end (SWM)
3. Width of pubic symphysis at the lower (caudal) end (SWL)

#### Pelvis AP (hip abduction view)

1. Width of pubic symphysis at the upper end (HWU)
2. Width of pubic symphysis at the midpoint of the upper and lower end (HWM)
3. Width of pubic symphysis at the lower end (HWL)

#### Pelvic CT, axial view

Measured level: where anterior border and posterior border can be seen (S1 level)

1. Width of pubic symphysis at the anterior border (AWA)
2. Width of pubic symphysis at the narrowest point (AWN)
3. Width of pubic symphysis at the widest point posterior to narrowest point (AWW)
4. Width of pubic symphysis at the posterior border (AWP)
5. Length from the anterior border to narrowest point (LAN)
6. Length from the narrowest point to widest point (LNW)
7. Length from widest point to the posterior border (LWP)
8. Thickness of the pubic symphysis in axial view (AT)

#### Pelvic CT, coronal view

Measured level: where just anterior the femur head begin to appear

1. Width of pubic symphysis at the upper end (CWU)
2. Width of pubic symphysis at the beginning of widening (CWW)
3. Width of pubic symphysis at the lower end (CWL)
4. Length from the upper end to the beginning of widening (LUW)
5. Length from beginning of widening to the lower end (LWL)
6. Thickness of the pubic symphysis in coronal view (CT)

This study adhered to the principles outlined in the Declaration of Helsinki. Informed consent was obtained from all subjects or, if subjects are under 18, from a parent and/or legal guardian.

The independent two-sample t-test was used for comparisons between males and females. Simple linear regression analysis was used to evaluate linear correlations among age groups. P values < .05 were considered to indicate statistical significance. SPSS version 25 (IBM Corp., Armonk, NY, USA) was used for statistical analysis.

## Results

### Pelvis AP, supine

Among the 784 films measured, 392 were males and 392 were females. Mean age was 56.6±18.9 years (range: 10–99) (Table 2).

**Table 2:** Patient demography and width of the pubic symphysis

Method	Measurement	N	Sex	Age	Mean±SD (mm)
Pelvis AP (supine)	SWU	784	392 M/392F	56.6±18.9	3.7±2.2
	SWM				4.7±2.0
	SWL				4.8±2.5
Pelvis AP (hip abduction)	HWU	280	144 M/136F	46.6±17.3	3.8±2.9
	HWM				4.6±2.3
	HWL				4.8±3.1
Pelvic CT (axial)	AWA	498	252 M/246F	55.25±18.9	15.0±6.2
	AWN				3.1±1.5
	AWW				4.1±1.6
	AWP				2.3±1.3
	TA				27.1±5.3
	LAN				7.0±2.4
	LNW				7.8±3.1
	LWP				12.4±4.7
Pelvic CT (coronal)	CWU	776	388 M/388F	56.7±18.9	3.1±4.1
	CWW				3.6±4.5
	CWL				20.5±8.2
	TC				20.4±7.1
	LUW				15.3±6.3
	LWL				5.0±2.9

*SWU* width of pubic symphysis at the upper end, *SWM* width of pubic symphysis at the midpoint of the upper and lower end, *SWL* width of pubic symphysis at the lower end, *HWU* width of pubic symphysis at the upper end, *HWM* width of pubic symphysis at the midpoint of the upper and lower end, *HWL* width of pubic symphysis at the lower end, *AWA* width of pubic symphysis at the anterior border, *AWN* width of pubic symphysis at the narrowest point, *AWW* width of pubic symphysis at the widest point posterior to the narrowest point, *AWP* width of pubic symphysis at the posterior border, *LAN* length from the anterior border to the narrowest point, *LNW* length from narrowest point to the widest point, *LWP* length from the widest point to the posterior border, *AT* thickness of the pubic symphysis in the axial view, *CWU* width of pubic symphysis at the upper end, *CWW* width of pubic symphysis at the beginning of widening, *CWL* width of pubic symphysis at the lower end, *LUW* length from the upper end to the beginning of widening, *LWL* length from the beginning of widening to the lower end, *CT* thickness of the pubic symphysis in the coronal view

#### Width of pubic symphysis at the upper end (SWU)

**Fig. 2:**
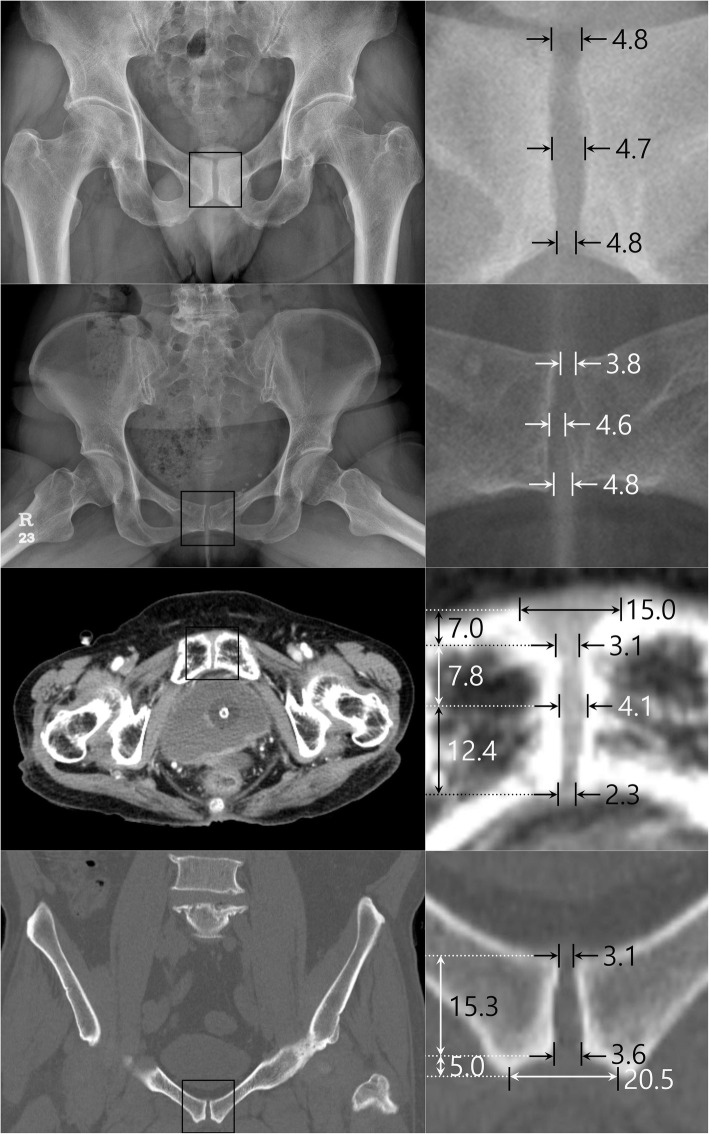
Mean width of the pubic symphysis. First raw: pelvis AP (supine), second raw: pelvis AP (hip abduction view), third raw: pelvic CT (axial view), fourth raw: pelvic CT (coronal view), unit: mm

**Fig. 3:**
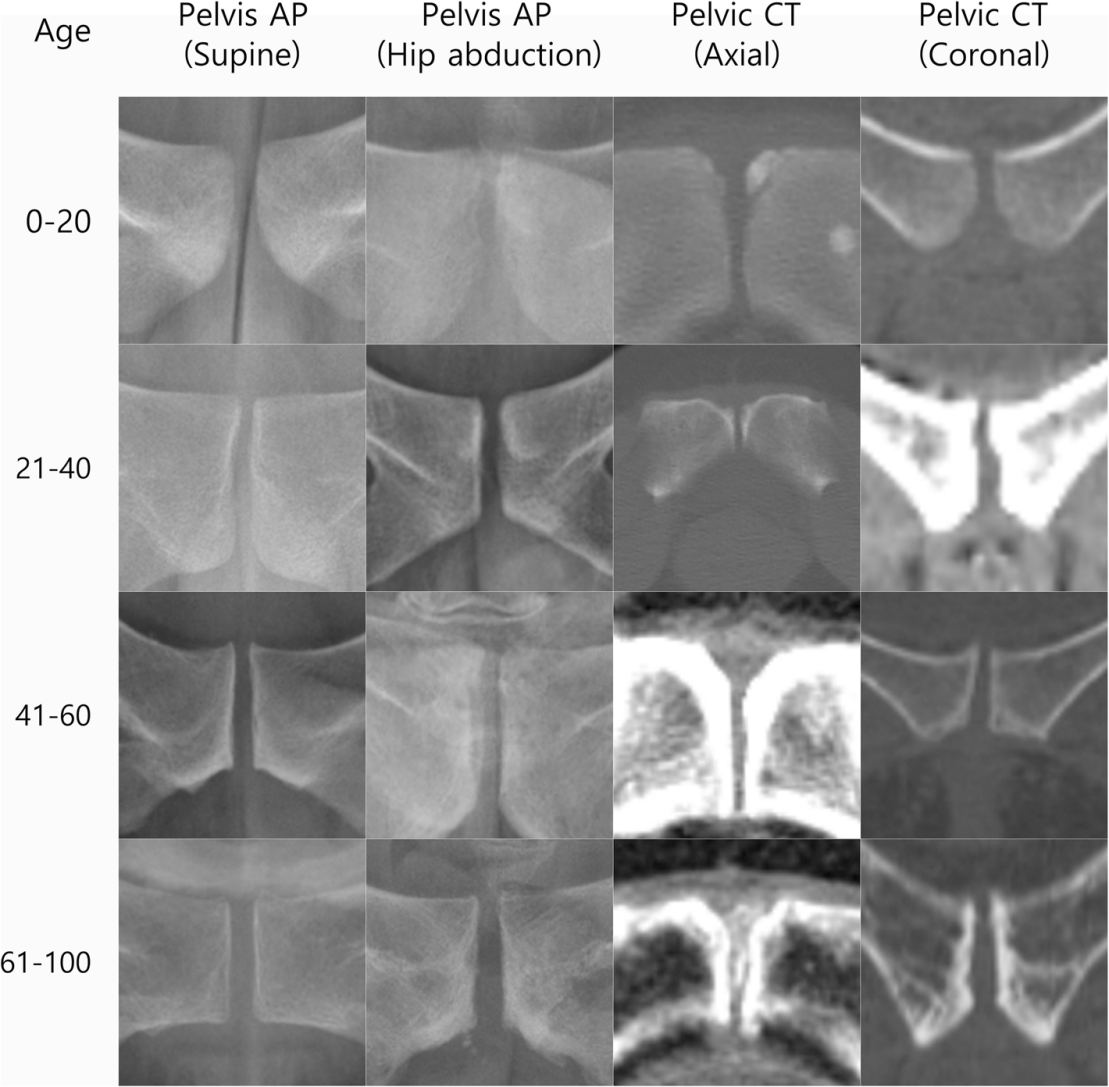
Width of pubic symphysis relating to age in each view. First column: pelvis AP (supine), second column: pelvis AP (hip abduction view), third column: pelvic CT (axial view), fourth column: pelvic CT (coronal view), first raw: 0–20 years, second raw: 21–40 years, third raw: 41–60 years, fourth raw: 61–100 years

Mean SWU was 3.7±2.2 mm (Fig. 2). SWU was significantly wider in females (4.04±2.76 mm) than males (3.46±1.38 mm, p=0.006) (Table 3). There were significant differences among the age groups (p < 0.001) (Table 4). SWU significantly increased with age (p=0.022) (Fig. 3).

**Table 3:** Width of pubic symphysis relating to sex in each view

Method	Measurement	Male (n=392)		Female (n=392)	p value
Pelvis AP (supine)	SWU	3.46±1.38	<	4.04±2.76	0.006
	SWM	4.64±1.58	<	4.75±2.29	< 0.001
	SWL	4.58±2.19	≒	5.08±2.76	0.251
		Male (n=144)		Female (n=136)	
Pelvis AP (hip abduction)	HWU	3.65±1.50	≒	3.97±3.85	0.179
	HWM	4.45±2.16	≒	5.18±3.79	0.251
	HWL	4.55±1.30	<	4.74±3.06	0.004
		Male (n=252)		Female (n=246)	
Pelvic CT (axial)	AWA	14.50±6.62	≒	16.44±6.22	0.285
	AWN	3.19±1.53	≒	3.09±1.50	0.523
	AWW	4.27±1.60	≒	4.00±1.50	0.786
	AWP	2.20±1.30	≒	2.44±1.40	0.265
	TA	29.48±4.60	≒	24.70±4.82	0.579
	LAN	7.22±2.46	≒	6.76±2.41	0.149
	LNW	8.13±3.36	>	7.42±2.79	0.016
	LWP	14.13±4.83	>	10.52±3.66	< 0.001
		Male (n=388)		Female (n=388)	
Pelvic CT (coronal)	CWU	2.28±1.26	<	3.83±5.48	< 0.001
	CWW	2.68±1.63	<	4.54±6.08	< 0.001
	CWL	17.49±4.53	<	23.60±9.86	< 0.001
	TC	24.50±5.98	≒	16.23±5.61	0.600
	LUW	18.59±5.40	≒	12.06±5.33	0.530
	LWL	5.91±3.03	>	4.16±2.39	0.007

*SWU* width of pubic symphysis at the upper end, *SWM* width of pubic symphysis at the midpoint of the upper and lower end, *SWL* width of pubic symphysis at the lower end, *HWU* width of pubic symphysis at the upper end, *HWM* width of pubic symphysis at the midpoint of the upper and lower end, *HWL* width of pubic symphysis at the lower end, *AWA* width of pubic symphysis at the anterior border, *AWN* width of pubic symphysis at the narrowest point, *AWW* width of pubic symphysis at the widest point posterior to the narrowest point, *AWP* width of pubic symphysis at the posterior border, *LAN* length from the anterior border to the narrowest point, *LNW* length from the narrowest point to the widest point, *LWP* length from the widest point to the posterior border, *AT* thickness of the pubic symphysis in axial view, *CWU* width of pubic symphysis at the upper end, *CWW* width of pubic symphysis at the beginning of widening, *CWL* width of pubic symphysis at the lower end, *LUW* length from the upper end to the beginning of widening, *LWL* length from the beginning of widening to the lower end, *CT* thickness of the pubic symphysis in coronal view; unit: mm, >: significantly greater, <: significantly lesser, ≒: no significant difference

**Table 4:** Width of pubic symphysis relating to age in each view

Method	Measurement	0–20 (n=16)	21–40 (n=162)	41–60 (n=253)	61–100 (n=353)	p value
Pelvis AP (supine)	SWU	6.1±2.1	3.4±1.5	3.4±1.4	4.1±2.7	< 0.001
	SWM	4.6±1.7	4.4±1.9	4.4±1.5	5.0±2.3	< 0.001
	SWL	9.4±3.8	5.4±3.1	4.3±1.7	4.8±2.4	< 0.001
		0–20 (n=10)	21–40 (n=109)	41–60 (n=98)	61–100 (n=63)	
Pelvis AP (hip abduction)	HWU	6.6±2.3	3.4±1.3	3.5±1.2	4.5±5.4	0.001
	HWM	4.5±1.1	4.3±1.5	4.5±1.5	5.5±4.0	0.010
	HWL	8.1±2.7	4.8±2.1	4.1±1.5	5.4±5.2	< 0.001
		0–20 (n=8)	21–40 (n=122)	41–60 (n=156)	61–100 (n=212)	
Pelvic CT (axial)	AWA	21.0±7.7	15.9±7.3	15.0±6.0	14.3±5.5	0.005
	AWN	4.2±1.8	3.0±1.5	2.9±1.4	3.3±1.6	0.020
	AWW	5.0±1.8	4.2±1.6	3.8±1.5	4.3±1.5	0.027
	AWP	3.9±2.4	2.4±1.4	2.1±1.2	2.3±1.4	0.002
	TA	26.0±6.2	27.1±4.9	27.5±5.3	26.9±5.5	0.720
	LAN	8.9±3.2	7.0±2.6	7.0±2.4	6.8±2.3	0.169
	LNW	6.7±2.2	7.6±3.2	8.4±3.4	7.5±2.8	0.029
	LWP	10.4±5.1	12.5±4.5	12.1±4.7	12.5±4.7	0.520
		0–20 (n=16)	21–40 (n=157)	41–60 (n=252)	61–100 (n=351)	
Pelvic CT (coronal)	CWU	3.9±1.4	2.6±1.5	2.5±1.8	3.6±5.7	0.002
	CWW	4.6±1.7	3.5±3.0	3.0±2.2	4.0±6.1	0.033
	CWL	18.2±4.5	18.1±5.9	20.3±7.2	21.9±9.6	< 0.001
	TC	18.1±3.4	19.3±6.2	20.6±6.9	20.8±7.7	0.085
	LUW	10.8±3.6	14.4±5.8	15.9±6.2	15.5±6.5	0.002
	LWL	7.2±2.6	4.9±3.1	4.7±2.2	5.2±3.1	0.002

*SWU* width of pubic symphysis at the upper end, *SWM* width of pubic symphysis at the midpoint of the upper and lower end, *SWL* width of pubic symphysis at the lower end, *HWU* width of pubic symphysis at the upper end, *HWM* width of pubic symphysis at the midpoint of the upper and lower end, *HWL* width of pubic symphysis at the lower end, *AWA* width of pubic symphysis at the anterior border, *AWN* width of pubic symphysis at the narrowest point, *AWW* width of pubic symphysis at the widest point posterior to the narrowest point, *AWP* width of pubic symphysis at the posterior border, *LAN* length from the anterior border to the narrowest point, *LNW* length from the narrowest point to the widest point, *LWP* length from the widest point to the posterior border, *AT* thickness of the pubic symphysis in axial view, *CWU* width of pubic symphysis at the upper end, *CWW* width of pubic symphysis at the beginning of widening, *CWL* width of pubic symphysis at the lower end, *LUW* length from the upper end to the beginning of widening, *LWL* length from the beginning of widening to the lower end, *CT* thickness of the pubic symphysis in coronal view; unit: mm

#### Width of pubic symphysis at the midpoint of the upper and lower end (SWM)

Mean SWM was 4.7±2.0 mm (Fig. 2). SWM was significantly wider in females (4.75±2.29 mm) than males (4.64±1.58 mm, p < 0.001) (Table 3). There were significant differences among the age groups (p < 0.001) (Table 4). SWM significantly increased with age ( p< 0.001) (Fig. 3).

#### Width of pubic symphysis at the lower end (SWL)

Mean SWL was 4.8±2.5 mm(Fig. 2). There was no significant difference between males (4.58±2.19 mm) and females (5.08±2.76 mm) (p=0.251) (Table 3). There were significant differences among the age groups (p < 0.001) (Table 4). SWL significantly increased with age (p < 0.001) (Fig. 3).

### Pelvis AP, abducted

Among the 280 films measured, 144 were males and 136 were females. Mean age was 46.6±17.3 years (range: 16–92) (Table 2).

#### Width of pubic symphysis at the upper end (HWU)

Mean HWU was 3.8±2.9 mm (Fig. 2). There was no significant difference between males (3.65±1.50 mm) and females (3.97±3.85 mm) (p=0.179) (Table 3). There were significant differences among the age groups (p=0.001) (Table 4). There was no significant difference with aging (p=0.223) (Fig. 3).

#### Width of pubic symphysis at the midpoint of the upper and lower end (HWM)

Mean HWM was 4.6±2.3 mm (Fig. 2). There was no significant difference between males (4.45±2.16 mm) and females (5.18±3.79 mm) (p=0.251) (Table 3). There were significant differences among the age groups (p=0.010) (Table 4). HWM significantly increased with age (p=0.003) (Fig. 3).

#### Width of pubic symphysis at the lower end (HWL)

Mean HWL was 4.8±3.1 mm (Fig. 2). HWL was significantly wider in females (4.74±3.06 mm) than males (4.55±1.30 mm) (p=0.004) (Table 3). There were significant differences among the age groups (p≤ 0.001) (Table 4). There was no significant difference with aging (p=0.574) (Fig. 3).

### Pelvis CT, axial view

Among the 498 films measured, 252 were males and 246 were females. Mean age was 55.25±18.9 years (range: 10–99) (Table 2).

#### Width of pubic symphysis at the anterior border (AWA)

Mean AWA was 15.0±6.2 mm (Fig. 2). There was no significant difference between males (14.50±6.62 mm) and females (16.44±6.22 mm) (p=0.285) (Table 3). There were significant differences among the age groups (p=0.005) (Table 4). AWA significantly increased with age (p=0.002) (Fig. 3).

#### Width of pubic symphysis at the narrowest point (AWN)

Mean AWN was 3.1±1.5 mm (Fig. 2). There was no significant difference between males (3.19±1.53 mm) and females (3.09±1.50 mm) (p=0.523) (Table 3). There were significant differences among the age groups (p=0.020) (Table 4). There was no significant difference with aging (p=0.285) (Fig. 3).

#### Width of pubic symphysis at the widest point posterior to narrowest point (AWW)

Mean AWW was 4.1±1.6 mm (Fig. 2). There was no significant difference between males (4.27±1.60 mm) and females (4.00±1.50 mm) (p=0.786) (Table 3). There were significant differences among the age groups (p=0.027) (Table 4). There was no significant difference with aging (p=0.791) (Fig. 3).

#### Width of pubic symphysis at the posterior border (AWP)

Mean AWP was 2.3±1.3 mm (Fig. 2). There was no significant difference between males (2.20±1.30 mm) and females (2.44±1.40 mm) (p=0.265) (Table 3). There were significant differences among the age groups (p=0.002) (Table 4). There was no significant difference with aging (p=0.094) (Fig. 3).

#### Thickness of the pubic symphysis in axial view (TA)

Mean TA was 27.1±5.3 mm (Fig. 2). There was no significant difference between males (29.48±4.60 mm) and females (24.70±4.82 mm) (p=0.579) (Table 3). There were significant no differences among the age groups (p=0.720) (Table 4). There was no significant difference with aging (p=0.141) (Fig. 3).

#### Length from anterior border to the narrowest point (LAN)

Mean LAN was 7.0±2.4 mm (Fig. 2). There was no significant difference between males (7.22±2.46 mm) and females (6.76±2.41 mm) (p=0.149) (Table 3). There were significant no differences among the age groups (p=0.169) (Table 4). There was no significant difference with aging (p=0.112) (Fig. 3).

#### Length from narrowest point to the widest point (LNW)

Mean LNW was 7.8±3.1 mm (Fig. 2). LNW was significantly wider in females (7.42±2.79 mm) than males (8.13±3.36 mm, p=0.016) (Table 3). There were significant differences among the age groups (p=0.029) (Table 4). There was no significant difference with aging (p=0.962) (Fig. 3).

#### Length from widest point to the posterior border (LWP)

Mean LWP was 12.4±4.7 mm (Fig. 2). LWP was significantly wider in males (14.13±4.83 mm) than females (10.52±3.66 mm, p< 0.001) (Table 3). There were significant no differences among the age groups (p=0.520) (Table 4). There was no significant difference with aging (p=0.639) (Fig. 3).

### Pelvis CT, coronal view

Among the 776 films measured, 388 were males and 388 were females. Mean age was 56.7±18.9 years (range: 10–99) (Table 2).

#### Width of pubic symphysis at the upper end (CWU)

Mean CWU was 3.1±4.1 mm (Fig. 2). CWU was significantly wider in females (3.83±5.48 mm) than males (2.28±1.26 mm, p < 0.001) (Table 3). There were significant differences among the age groups (p< 0.001) (Table 4). CWU significantly increased with age (p < 0.001) (Fig. 3).

#### Width of pubic symphysis at beginning of widening (CWW)

Mean CWW was 3.6±4.5 mm (Fig. 2). CWW was significantly wider in females (4.54±6.08 mm) than males (2.68±1.63 mm, p < 0.001) (Table 3). There were significant differences among the age groups (p < 0.001) (Table 4). CWW significantly increased with age (p=0.012) (Fig. 3).

#### Width of pubic symphysis at the lower end (CWL)

Mean CWL was 20.5±8.2 mm (Fig. 2). CWL was significantly wider in females (23.60±9.86 mm) than males (17.49±4.53 mm, p < 0.001) (Table 3). There were significant differences among the age groups (p < 0.001) (Table 4). CWL significantly increased with age (p< 0.001) (Fig. 3).

#### Thickness of the pubic symphysis in coronal view (TC)

Mean TC was 20.4±7.1 mm (Fig. 2). There was no significant difference between males (24.50±5.98 mm) and females (16.23±5.61 mm) (p=0.600) (Table 3). There were no significant differences among the age groups (p=0.600) (Table 4). There was no significant difference with aging (p=0.055) (Fig. 3).

#### Length from the upper end to the beginning of widening (LUW)

Mean LUW was 15.3±6.3 mm (Fig. 2). There was no significant difference between males (18.59±5.40 mm) and females (12.06±5.33 mm) (p=0.530) (Table 3). There were significant differences among the age groups (p=0.530) (Table 4). LUW significantly increased with age (p=0.039) (Fig. 3).

#### Length from the beginning of widening to the lower end (LWL)

Mean LWL was 5.0±2.9 mm (Fig. 2). LWL was significantly wider in males (5.91±3.03 mm) than females (4.16±2.39 mm, p=0.007) (Table 3). There were significant differences among the age groups (p=0.007) (Table 4). There was no significant difference with aging (p=0.805) (Fig. 3).

### Comparison of pelvis AP and pelvis CT

Width of pubic symphysis at the upper end (WU) of supine film differed significantly from that of hip abduction film or coronal CT (p < 0.001). Coronal CT was lesser than supine film and hip abduction film.

Width of pubic symphysis at the lower end (WL) of supine film differed significantly from that of hip abduction film or coronal CT (p< 0.001). Coronal CT was greater than supine film and hip abduction film.

## Discussion

In supine pelvis AP, the width of pubic symphysis significantly increased with age at the upper end and midpoint (SWU: p=0.022, SWM: p< 0.001). However, the width of pubic symphysis significantly decreased with age at the lower end (SWL: p< 0.001). In hip abduction pelvis AP, the width of pubic symphysis at the midpoint (HWM) significantly increased with age (p=0.003).

In axial CT, the width of pubic symphysis at the anterior border (AWA) significantly decreased with age (p=0.002). However, the width of pubic symphysis at the posterior border (AWP) did not change significantly with age (p=0.094). In coronal CT, like supine film, the width of pubic symphysis significantly increased with age at its entire length (upper end, CWU: p< 0.001; beginning of widening CWW: p=0.012; and lower end, CWL: p< 0.001). Also, the length from the upper end to the beginning of widening (LUW) significantly increased with age (p=0.039).

The pelvic malunion has been defined as greater than 5-mm of displacement of the hemipelvis and pubic symphysis in a nonanatomic position, whether in a rotational or translational fashion [6].

Since the width of pubic symphysis significantly increased with age at its entire length, we do think that pelvic malunion should be defined according to the population and age.

The fixation failure is thought to be due to the intimate relationship between the adductor longus; rectus abdominis; and symphyseal cartilage, disk, and capsular tissues. The adductor longus and rectus abdominis are attached to the capsule and disk of the pubic symphysis. All adductor tendons are attached to the pubis [5].

A study invested structural organization of the mineralized cartilage of human pubic symphysis and found mineralization of cartilage is intermittent from 20 to 29 years, amount of gaps becomes less by 40–49 years and becomes intermittent again at the age of 70–79 years. In elderly and senile people, mineral plates of complex configuration appear in the interterritorial matrix and chondrocyte capsules; by the age of 87, there appear thick highly mineralized bundles of collagen fibers [10]. It is notable that in middle-age group, the amount of gap becomes less coincides well with our results (4.0±1.6 mm, lesser than 21–40 group or 61–100 group) (Supplement Table 1).

Mean widths determined by imaging studies of 130 non-pregnant women yielded 2.6 mm [11]. While 12.58±4.48 mm was measured at the most anterior part of the joint in women who had on average given birth to three children [12]. Alicioglu, in the single CT study, did not find any relationship between symphyseal width and parity or body mass index [12].

In a study of adult cadavers, Loeschcke (1912) calculated mean joint widths to be 4.9±1.3 mm in men, 7.5±4.1 mm in nulliparous women, and 20.0±3.8 mm in multiparous women [7].

Comparing our X-ray data with Loeschcke, Caucasians (German) women have wider pubic symphysis than Korean women (p< 0.001). However, there was no significant difference between Caucasian men and Korean men (p=0.0951).

Comparing the bony pelvis of European American women (EA) and Korean women (Kor) from the literatures [13, 14], European women have larger interspinous distance (EA: 104±9 mm, Kor: 94.0±7.2 mm, p< 0.001) and intertuberous distance (EA: 133.5±9.6 mm, Kor: 97.7±10.1 mm, p< 0.001) than Koreans. From this, it is thought that the wider pubic symphysis in German women than Korean women is due to the larger pelvis size of Germans.

Comparing our CT data with Alicioglu [12], Koreans have a wider anterior border (p< 0.001) but, however, narrower posterior border (p< 0.001) than Turkish (Table 5). In this study, we could see that Koreans have a narrower pubic symphysis than Caucasians.

**Table 5:** Comparison of the width of the pubic symphysis in different ethnic groups

Author (Year)	Ethnic	Measurement			
		X-ray	CT	USG	P
Hwang (present study)	Korean	M: 4.2±1.8 F: 4.6±2.6 Total: 4.4±2.3	(Total) AWA: 15.0±6.0 AWP: 2.3±1.3 (Female) AWA: 16.4±6.2 AWN: 3.1±1.5		
Loeschcke (1934)	German	M: 4.9±1.3 F: 7.5±4.1 Pregnancy: 21.1±4.3 Multiparous: 20.0±3.8			M: 0.095 F: < 0.001
Roberts (1934)	British	(Non-pregnant) Nulliparae: 2.6 Parae: 2.6 (Pregnant) Primigravidae: 4.2 Multiparae: 5.0			
Aligioglu (2008)	Turkish		AWA: 12.2±1.2 AWP: 3.7±4.0		AWA, AWP: <0.001
Becker (2014)	New Zealanders			(Nulliparous) AWA:10.1±4.9 AWN: 2.6±0.7	AWA: <0.001 AWN: 0.071

*CT* computed tomography, *USG* ultrasonography, *P* p value, each p value is compared with the present study, *M* male, *F* female, *AWA* width of pubic symphysis at the anterior border, *AWP* width of pubic symphysis at the posterior border, *AWN* width of pubic symphysis at the narrowest point

In Korean women (CT measured), the width of pubic symphysis at the narrowest point (3.1±1.5 mm) did not differ to that of New Zealand nulliparous women (USG measured, 2.6±0.7 mm) significantly (p=0.071). However, Korean women (16.4±6.2 mm) have a significantly wider width of pubic symphysis at the anterior border than New Zealand nulliparous women (10.1±4.9 mm, p< 0.001) (Table 5) [15].

Recently, the elastic band has been used in the management of obstetric pubic symphyseal separation. Once the elastic band device was in place, on postpartum day 1, radiography showed a decrease of the pubic width from 41 to 12 mm. Use of an elastic band device was associated with a reduction of the pubic width and pain associated after obstetric pubic symphysis separation [16]. Our present data can be a baseline to the evaluation of the effect of the management of pubic diastasis as obstetric separation.

In the present study, we did not analyze the inter-observer and intra-observer errors. However, two experienced researchers with such image assessment (two surgeons) were involved in order to reduce these errors [17]. We could not analyze the parity of the women (nulliparous or multiparous) included, and this is the limitation of the study.

Pelvic malunion should be defined according to the population and age. The results of this study can be a practical reference in assessing the quality of reduction after internal fixation of the patients with traumatic diastasis of the pubic symphysis. Further study is needed to introduce a new guideline for the pelvic diastasis according to age, sex, and population.

## Supplementary Information

**Additional file 1:.** Supplemental Table 1. Comparison of age-related change of pubic symphysis.

## Abbreviations

SWU: Width of pubic symphysis at upper (cranial) end
SWM: Width of pubic symphysis at the midpoint of upper and lower end
SWL: Width of pubic symphysis at the lower (caudal) end
HWU: Width of pubic symphysis at the upper end
HWM: Width of pubic symphysis at the midpoint of upper and lower end
HWL: Width of pubic symphysis at the lower end
AWA: Width of pubic symphysis at the anterior border
AWN: Width of pubic symphysis at the narrowest point
AWW: Width of pubic symphysis at the widest point posterior to the narrowest point
AWP: Width of pubic symphysis at the posterior border
LAN: Length from anterior border to the narrowest point
LMW: Length from narrowest point to the widest point
LWP: Length from the widest point to the posterior border
AT: Thickness of the pubic symphysis in axial view
CWU: Width of pubic symphysis at the upper end
CWW: Width of pubic symphysis at the beginning of widening
CWL: Width of pubic symphysis at the lower end
LUW: Length from the upper end to the beginning of widening
LWL: Length from the beginning of widening to the lower end
CT: Thickness of the pubic symphysis in coronal view
