# Supplementary material for: Width of pubic symphysis relating to age and sex in Koreans
Source: J Orthop Surg Res. 2021 Jul 3;16:430. doi: 10.1186/s13018-021-02561-9 (PMC8254372; doi:10.1186/s13018-021-02561-9)
Supplement: Supplementary file 1 — Additional file 1:. Supplemental Table 1. Comparison of age-related change of pubic symphysis. [file 13018_2021_2561_MOESM1_ESM.doc]

Supplemental Table 1. Comparison of age-related change of pubic symphysis

| Hwang (2018) | | |  | Neznakomtseva (1993) | | |
| --- | --- | --- | --- | --- | --- | --- |
| Age | N | X-Ray (mm) |  | Age | N | Ultramicroscopic levels |
| 0-20 | 16 | 6.7±3.3 |  |  |  |  |
| 21-40 | 162 | 4.4±2.4 |  | 20-29 | 15 | The front of mineralization of the pubic symphysis cartilage is intermittent. |
| 41-60 | 253 | 4.0±1.6 |  | 40-49 | 16 | The mount of gaps becomes less. |
| 61-100 | 353 | 4.6±2.5 |  | 70-79 | 20 | The front of mineralization becomes intermittent again. |
|  |  |  |  | 80-87 | 8 | There appear thick highly mineralized bundles of collagen fibers. |
